# Research on smoke control for an underground mall fire, based on smoke barrier and mechanical smoke exhaust system

**DOI:** 10.1038/s41598-022-16067-9

**Published:** 2022-07-29

**Authors:** Jinzhang Jia, Xiuyuan Tian, Fengxiao Wang

**Affiliations:** 1 College of Safety Science and Engineering, Liaoning Technical University, Fuxin, 123000 Liaoning China; 2 Ministry of Education, Key Laboratory of Mine Thermal Power Disaster and Prevention, Fuxin, 123000 Liaoning China

**Keywords:** Environmental sciences, Engineering

## Abstract

This study examines smoke spread in an underground mall fire under the composite smoke control mode of a smoke barrier and a mechanical smoke exhaust system. The objective is to optimize the selection of smoke containment and exhaust methods in an underground mall in Fuxin City, China. A Fire Dynamics Simulator was used for numerical simulation to investigate the effects of the sagging height and spacing of smoke barriers on smoke containment, as well as the effects of size, number, and arrangement of smoke vents in the mechanical smoke exhaust system on the effectiveness of smoke exhaust. The results indicated that a smoke barrier with a sagging height of 1 m and a spacing of 5 m was effective in preventing the spread of high-temperature smoke. When the sagging height of the smoke barrier increased to 1.2 m, the smoke barrier effect was comparable to that of a 1 m height barrier. Regarding the mechanical smoke exhaust system, the size of the opening area of the smoke vent had no significant effect on the smoke exhaust effect. The best smoke exhaust effect was achieved when the number of smoke vents was 12. Additionally, the double-row setting of smoke vents was more efficient than the single-row setting. Combining a smoke barrier and a mechanical smoke exhaust system can provide a more effective smoke control compared to either system alone. Comprehensively, considering the effectiveness and economy of smoke containment and exhaust, the optimal combination of smoke containment and exhaust was determined to be a smoke barrier with a sagging height of 1 m and spacing of 5 m, and a mechanical smoke exhaust system with 12 smoke vents in a double-row arrangement.

## Introduction

Although the emergence of underground shopping malls has provided great convenience for citizens, underground buildings often have worse fire safety compared with the aboveground buildings. In case of fire in an underground building, common problems include high internal temperature, large amount of smoke due to poor ventilation conditions, poor efficiency in evacuating occupants, and difficulty in extinguishing the fire, all of which pose great threats to human safety. High-temperature smoke is the biggest obstacle to safe evacuation, as it can restrict visibility and cause burns^1–3^. Therefore, great attention should be paid to mitigating the spread of smoke in underground shopping malls.

The smoke barrier has been widely used in China and other parts of the world as an effective method to stop the spread of smoke^4–7^. Zheng et al.^8^ studied the effect of an automatic smoke barrier on the smoke flow of tunnel fires and conducted a simulation test study through the fire simulation software Fire Dynamics Simulator (FDS). The results indicated that the automatic smoke barrier had a good control effect on the smoke diffusion and temperature distribution in the tunnel during a fire, and it reduced the interference of lateral wind speed on upper smoke and smoke backflow, suggesting it can be used as an effective method to control tunnel fires. Several studies have shown that a well-designed mechanical smoke extraction system, which actively removes smoke, can reduce heat by 80% during a fire^9–12^. Ran et al.^13^ combined natural and mechanical ventilation mechanisms to analyze the effect of hybrid ventilation on reducing carbon monoxide (CO) concentrations and suppressing the horizontal diffusion of smoke. The results indicated that the average CO concentration in the concourse decreased significantly with the increase of roof window size and air exchange rate in the subway station, whereas the CO concentration in the atrium did not change significantly. Furthermore, hybrid ventilation was more effective in suppressing smoke diffusion compared with conventional mechanical ventilation. Yuan et al.^14^ studied the effect of mechanical smoke vent location and mechanical smoke extraction rate on the smoke extraction effect during fires in underground malls. The results indicated that for underground malls with ceiling partitions limiting smoke exhaust, mechanical smoke exhaust worked better when the smoke vents were located on the right wall rather than on the ceiling.

Many researchers have proposed the use of mechanical smoke exhaust system in conjunction with smoke barrier to achieve effective control of smoke spread in a short period of time and to extend the effective evacuation time in case of fire^15,16^. For example, Li et al.^17^ utilized numerical simulation and full-size promenade test methods to conduct a series of studies on the effect of the combination of smoke barrier with mechanical smoke exhaust and air curtain. Their results showed that this composite smoke control mode can effectively improve the efficiency of mechanical smoke exhaust and reduce its downstream flue gas temperature. Chen et al.^18^ investigated the smoke spread law with the composite smoke control mode of air curtain and smoke exhaust system during tunnel fires. Specifically, they used FDS to perform numerical simulation to investigate the effects of jet velocity, smoke exhaust volume, and spacing between air curtain and smoke vent on smoke containment and exhaust. The results indicated that the spacing between the air curtain and smoke vent had a strong influence on the jet characteristics and smoke spread, and the smoke control effect was best with a spacing of 30 m. The optimal smoke containment and smoke exhaust was achieved with 20 m/s jet velocity and 100 m^3^/s smoke exhaust volume.

In the past, scholars have used both smoke barriers and mechanical smoke exhaust systems or a combination of both to control the spread of smoke from fires in tunnels, subway stations, high-rise buildings, etc. However, studies on underground mall fires have mostly been limited to smoke control systems composed only of smoke barriers rather than mechanical smoke exhaust systems or a combination of smoke barriers and mechanical smoke exhaust systems. Therefore, based on the previous research, numerical simulation methods are used in the present study to investigate the influence of the sagging height and spacing of smoke barriers on the smoke containment performance of smoke barriers, as well as to investigate the influence of the size, number, and arrangement of smoke vents on the smoke exhaust performance of mechanical smoke exhaust system. The results are then used to optimize the combination of smoke barriers and a mechanical smoke exhaust system for a composite smoke control mode. Therefore, this study provides a framework for the selection of smoke barrier and smoke vent design parameters for smoke control in underground mall fires.

## Numerical simulation model

### Introduction to underground mall model

**Figure 1:**
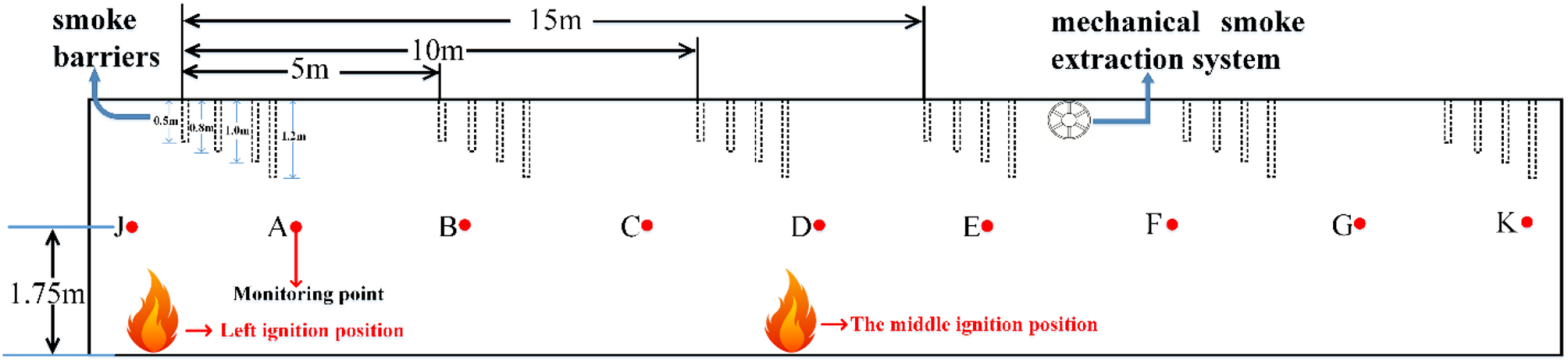
Plan of section 9–12 of underground commercial street.

Sections 9, 10, 11, and 12 of Fuxin city underground commercial street, which comprise an underground shopping mall, are located in the middle of Fuxin underground commercial street. His paper mainly simulates fire in this underground shopping mall. The effective utilization space of the shopping mall is 90 m long, 18.4 m wide, and 3 m high^19^. There are two exits on each side of the mall and each exit is 4.4 m wide and 2.7 m high, and each shop is 6.6 m wide. The schematic diagram of underground shopping mall model is shown in Fig. 1. In order to simplify the model, a stairway leading aboveground is established only for the exit at the upper left, whereas the other three exits are assumed to be directly connected to ground level.

### Setting of fire source and monitoring point

In order to study the fire smoke flow near the exit and the fire smoke flow in the middle of the corridor with high-risk coefficient, two representative fire source locations are selected with reference to the building plane structure, which are respectively on the left and middle of the corridor, and the fire source size is 2.0 m × 2.0 m, as shown in Fig. 1. According to the reference^20,21^ and the grid cell size of the geometric model of this simulation, the smoke temperature below 60 °C and visibility above 10 m at the characteristic height of human eye of 1.75 m are selected as the standards for safe evacuation. According to this standard, a total of 7 monitoring points (A, B, C, D, E, F and G) are set at an interval of 11 m in the shopping mall corridor, which are 1.75 m away from the ground and monitoring points (J and K) are set at the exit channel to monitor the change law of flue gas parameters. The heat release rate curve of the fire source growth stage is t^2^ type, the fire load density is set to 635 MJ/m^2^, the fire growth coefficient is 0.125, the maximum heat release rate is set to 5 MW, and the growth time is 200 s^22^.

### Grid settings

The space size to be divided into grids is an underground shopping mall with a size of 90 m × 18 m × 3 m and a stairwell of 18 m × 6 m × 10 m. The formula for the characteristic diameter D^*^ of the fire source is^23^

1 $$ D^{*} = \left( {\frac{{\dot{Q}}}{{\rho_{\infty } c_{p} T_{\infty } \sqrt g }}} \right)^{{2}/{5}}, $$

where: $\dot{Q}$—Heat release rate of fire source, kW; $c_{\infty }$—Ambient air density, kg·m^−3^; $c_{p}$—Specific heat of air at constant pressure, kJ·(kg K)^−1^; $T\infty$—Ambient air temperature, K; $g$—Gravitational acceleration, 9.81 m s^−2^.

If the ambient temperature is 293 K, the characteristic diameter of the 5 MW fire source will be 1.84 m. In order to ensure simulation accuracy, the ratio of $D^{*}$ to the grid cell size should be between 4 and 16^23^. The larger the ratio, the finer the simulation results and the longer the simulation time. The simulation’s accuracy and time consumption are considered comprehensively. The model is divided into three parts in which the number of grid cells is set to 270 × 50 × 8, 54 × 15 × 8, and 54 × 15 × 18, and the grid cell size is about 0.33 m × 0.4 m × 0.38 m, which is the same as the grid cell size. The ratio is between 4.6 and 5.6, which can meet the accuracy requirements.

### Boundary condition

The boundary condition of the smoke vent is set to exhaust, and the start mode of the smoke vent is set to start after the response of the temperature controller. The ambient temperature is 293 K, and the boundary at the outlet is set to ‘Open’.

### Simulation time

According to the construction area and exit width, and according to the regulations in^24^ and^25^, the area conversion coefficient of the ground floor is 0.7, and the average personnel density is 0.8 persons/m^2^. The average walking speed of people is 1.1 m/s, the average effective width reduction value of stairs and exits is 0.3 m, the fire detection time is 30 s, and the average response time of people is 120 s. The approximate calculation results show that the evacuation time of people in the worst case does not exceed 530 s. Therefore, the simulation time is set for 600 s.

## Results analysis and discussion

### Impact analysis of smoke barrier

#### Analysis of the impacts of the sagging height of smoke barrier

As shown in Fig. 1, taking the fire source on the left as an example, smoke barriers with four different sagging heights of 0.5 m, 0.8 m, 1.0 m, and 1.2 m are considered, and the smoke barriers are evenly distributed in the mall promenade at 5 m intervals^4,26^. For the fire simulation, there is no smoke exhaust system or automatic sprinkler system. From the simulation results, the law of the effect of the smoke barrier on smoke containment is obtained.

1. Height of smoke layer at each monitoring point
  Comparing the fire simulation results of different drop height smoke barriers in Fig. 2, we find that the smoke containment performance is significantly enhanced as the sagging height of the smoke barrier increases. With the smoke barrier sagging height of 0.5 m, at monitoring points A, B, C, D, E, and J, the smoke layer height changes in 34 s, 59 s, 82 s, 107 s, 174 s, and 43 s, respectively, whereas at monitoring points F, G, and K, smoke layer height was not detected changes. With the smoke barrier sagging height of 0.8 m, at monitoring points A, B, C, and J, the time of change of smoke layer height was 42 s, 70 s, 122 s, and 44 s, respectively, whereas at monitoring points D, E, F, G, and K, smoke layer height was not detected changes. With the smoke barrier sagging height of 1 m, at monitoring points A, B, C, and J, the smoke layer heights changed at 46 s, 85 s, 163 s, and 47 s, respectively, whereas at monitoring points D, E, F, G, and K, no change was detected. With the smoke barrier sagging height of 1.2 m, at monitoring points A, B, C, and J, for the change of smoke layer height was 46 s, 85 s, 167 s, and 47 s, respectively, whereas at monitoring points D, E, F, G, and K, for the change of smoke layer height was not detected. It can be seen that as the height of the smoke barrier increases, the height of the smoke layer at monitoring points B, C, D, E and J increases, and the time when the height of the smoke layer begins to decrease is gradually delayed. Compared with other monitoring points, the height of the smoke layer at monitoring point A tends to decrease with the increase in the height of the smoke barrier, mainly due to the closest distance to the fire source.

**Figure 2:**
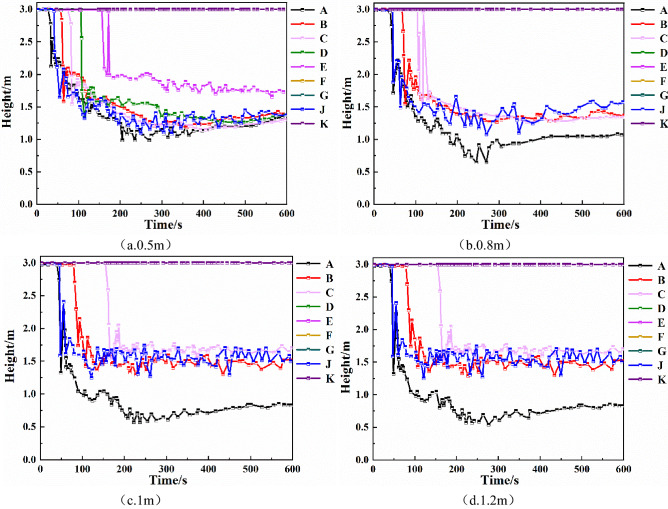
Smoke layer height at each monitoring point of smoke barrier with different sagging heights.
  Therefore, increasing the height of the smoke barrier can help to slow down the spread of smoke far from the fire source. When a fire occurs, you should escape from the fire source location as soon as possible. Among them, when the height of the smoke barrier reaches 1.2 m, the smoke-blocking effect is equivalent to that of 1 m.
2. Temperature of each monitoring point
  Figure 3 shows the comparison of the smoke temperature at each monitoring point under different sagging heights of the smoke barrier. As shown in the Fig. 3, the maximum temperature of monitoring point A increased with the increase of sagging heights of the smoke barrier, from 315 to 672 °C, mainly due to the closest location to the fire source. The maximum temperature of monitoring point D was below 50 °C when the smoke barrier was 0.5 m, and when the smoke barrier was 0.8 m, 1.0 m, and 1.2 m, the maximum temperature dropped to 20 °C. The temperature of the monitoring points E, F, G, and K did not change, and they were all at room temperature of 20 °C. For monitoring points B, C, and J, when the smoke barrier was 0.5 m, the maximum temperature was below 141 °C, 88 °C, 247 °C. When the smoke barrier was 0.8 m, the maximum temperature was 129 °C, 58 °C, and 229 °C. When the smoke barrier was 1.0 m, the maximum temperature of monitoring points B, C, J were respectively below 116 °C, 49 °C, 204 °C, when the smoke barrier was 1.2 m, the maximum temperature was below 116 °C, 49 °C, and 204 °C, respectively.

**Figure 3:**
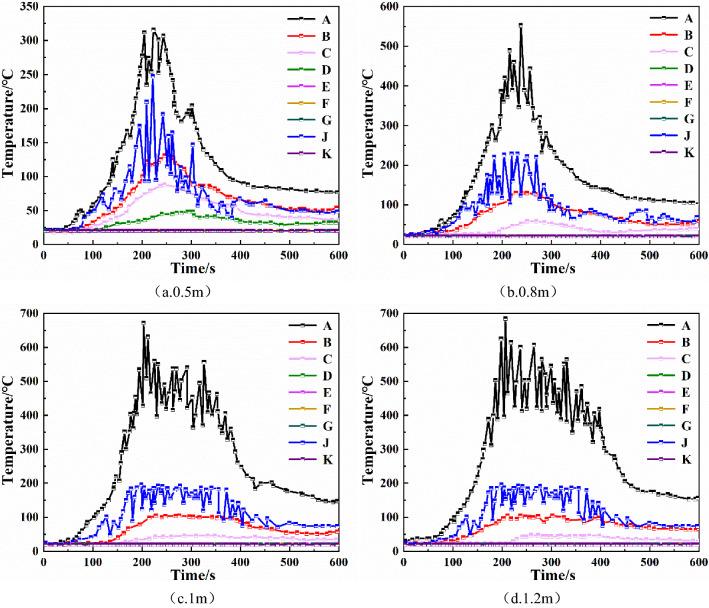
Smoke temperature at each monitoring point of smoke barrier with different sagging heights.
  It can be seen that the temperature at each monitoring point did not change significantly when the sagging height of the smoke barrier was changed from 1 to 1.2 m. The above showed that increasing the height of the smoke barrier to 1 m can effectively block the spread of high-temperature smoke far from the fire source, and the temperature of the monitoring point closer to the fire source would be higher. Thus, it is important to stay away from the fire source as soon as possible after the fire occurs.
3. Visibility of each monitoring point
  By comparing the visibility of each monitoring point in Fig. 4, it can be seen that increasing the sagging height of the smoke barrier in the range of 0.5–1 m is beneficial to improving the visibility monitoring point C, D, E in the strip underground mall. For monitoring points A, B, and J, which are closer to the fire source, increasing the sagging height of the smoke barrier reduced its visibility. When the sagging height of the smoke barrier is increased from 1 to 1.2 m, there is no significant enhancement in the visibility of each monitoring point.

**Figure 4:**
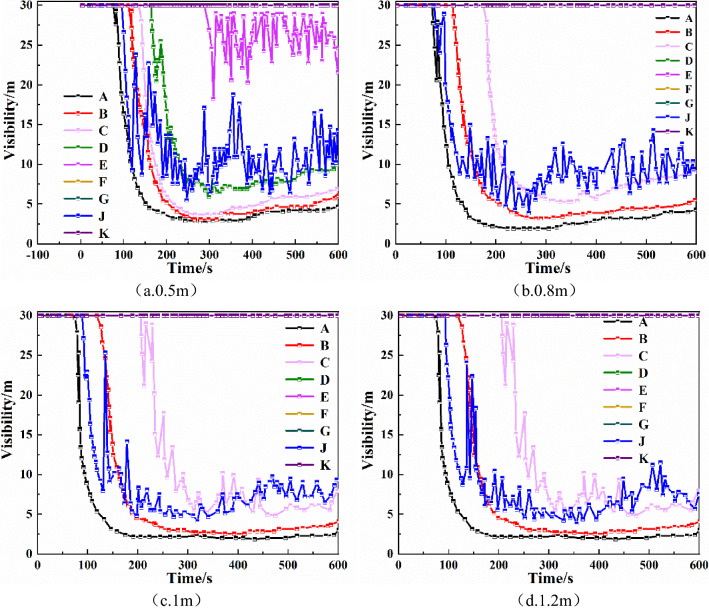
Visibility at each monitoring point of smoke barrier with different sagging heights.
  Taking the left fire source as an example, within the range of 0.5–1 m of the sagging height of the smoke barrier, the higher the sagging height of the smoke barrier, the higher the maximum temperature of the monitoring point nearer to the fire source, the greater the height drop of the smoke layer, and the lower the visibility. The farther away from the fire source, the lower the temperature of the monitoring point, the higher height of smoke layer and the higher the visibility. Therefore, once a fire begins, due to the role of the smoke barrier to prevent the spread of smoke from the fire source, occupants must quickly move away from the fire source. When the sagging height of the smoke barrier exceeds 1 m, which is 1.2 m, the smoke-blocking effect is equivalent to 1 m.

#### Analysis of the impact of placement spacing of smoke barrier

With the sagging height of the smoke barrier fixed at 1 m, three different smoke barrier spacings are tested, namely 15 m, 10 m, and 5 m^27^. For the simulation, there is no smoke exhaust system or automatic sprinkler system. The differences between the height, temperature, and visibility of the smoke layer at each monitoring point under different conditions are simulated and analyzed to determine the effect of smoke barrier spacing on smoke containment performance.

1. Height of smoke layer at each monitoring point
  As can be seen from Fig. 5, when the smoke barrier with a sagging height of 1 m is spaced at 15 m intervals, the smoke spreads all the way to monitoring point G. At monitoring points A, B, C, D, E, and J, the height of the smoke layer tends to fluctuate smoothly at about 240 s, and the final stable smoke layer height is below 1.5 m. At monitoring point F, the height of the smoke layer is in constant fluctuation, although the overall height is within a safe range, and at monitoring points G and K, the smoke layer height is relatively high. When the smoke barrier with a sagging height of 1 m is spaced at 10 m intervals, the smoke spreads to monitoring point E. At monitoring points A, B, C, D, and J, the smoke layer height tends to stabilize at about 180 s, and the stable height is below 1.5 m. At monitoring points A and B, the smoke layer height is the lowest, stabilizing at below 1 m, and at monitoring point E, the smoke layer height quickly falls to about 0.5 m within 180 s, then increases to about 2 m from 180 s to about 240 s, and finally fluctuates between 1.5 and 2 m. When the smoke barrier with sagging height of 1 m is spaced at 5 m intervals, the smoke spreads only to monitoring point C. The time when the smoke layer begins to fall at each monitoring point is greatly delayed, and the height of the smoke layer increases at each monitoring point except for monitoring point A.

**Figure 5:**
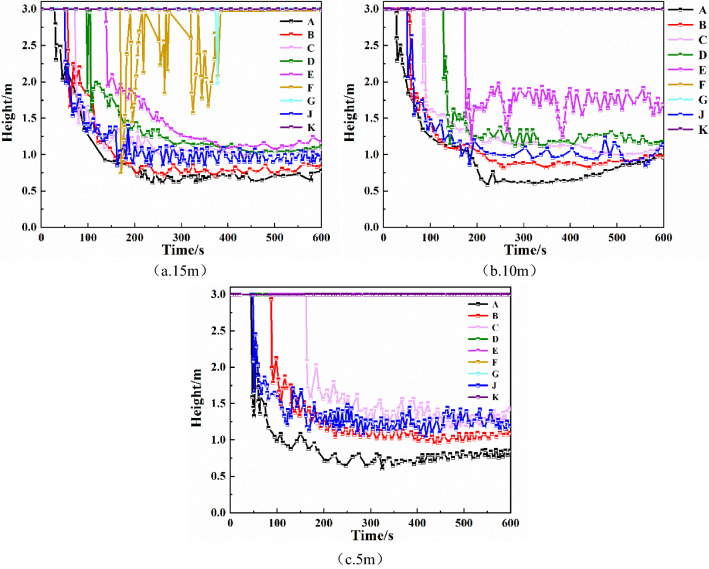
The height of the smoke layer at each monitoring point of the smoke barrier with different placement spacing.
  It can be seen that, in the case of the same fire source, the same sagging height of the smoke barrier, and the same environmental factors, when the smoke barriers are spaced more densely, the spread of smoke far from the fire source is slower and the height of the smoke layer at each monitoring point is greater, so the smoke containment effect is better.
2. Temperature of each monitoring point
  As can be seen from Fig. 6, as smoke barrier spacings decreases from 15 to 5 m, the temperature of monitoring point A gradually increases, and the maximum temperature increases from 500 to 670 °C. Monitoring points G and K are far away from the fire source, the smoke does not spread to this monitoring point, and the temperature has been maintained at room temperature of about 20 °C. The maximum temperatures of the monitoring points B, C, D, E, F and J all decrease with the decrease of smoke barrier spacings, and the maximum temperatures of the smoke barrier set at 15 m intervals are 312 °C, 204 °C, 79 °C, 48 °C, 36 °C and 307 °C respectively; the maximum temperatures of the smoke barrier set at 10 m intervals are 260 °C, 121 °C, 59 °C, 20 °C, 20 °C and 280 °C respectively, at this time E and F The temperature at the monitoring point is 20℃, which means that the smoke has not spread to the monitoring points of E and F under this condition; the maximum temperatures of the smoke barrier set at 5 m intervals are 143 °C, 47 °C, 20 °C, 20 °C, 20 °C, 20 °C, 249 °C, and the smoke has not spread to the monitoring points of D, E and F under this condition.

**Figure 6:**
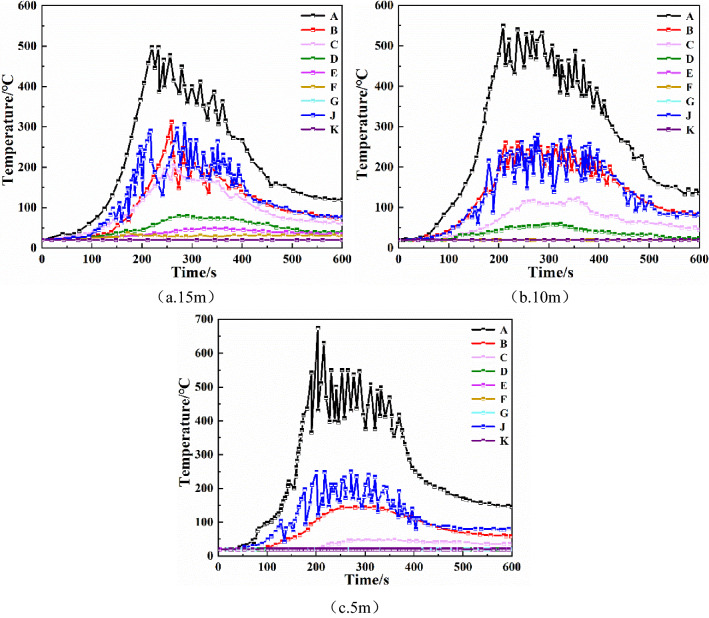
The temperature of each monitoring point of the smoke barrier with different spacing.
  It can be seen that shortening the spacing between smoke barriers increased the temperature at monitoring point A, which was closest to the fire source. For monitoring points far from the fire source, it greatly reduced temperature and provided favorable conditions for personnel to escape.
3. Visibility of each monitoring point
  As shown in Fig. 7, when the smoke barrier with sagging height of 1 m is spaced at 15 m intervals, the order of decreasing visibility of each monitoring point is A, C, J, B, D, E. The visibility at monitoring points F, G, and K does not change. At monitoring points A, C, J, B, D, and E, visibility begins to decline at 81 s, 101 s, 102 s, 118 s, 156 s, and 209 s, respectively. At monitoring point E, visibility stabilizes below 7 m, and at monitoring points A, C, J, B, and D, visibility is below 5 m. When the smoke barrier with sagging height of 1 m is spaced at 10 m intervals, the order of decreasing visibility of each monitoring point is A, B, J, C, D, and E, and the visibility of other monitoring points exhibits no change. At monitoring points A, B, J, C, D, and E, visibility begins to decline at 68 s, 93 s, 103 s, 125 s, 160 s, and 193 s, respectively. At monitoring point E, visibility changes only part of the time, and visibility is relatively high. The visibility at monitoring points A, B, J, C, and D finally stabilizes below 5 m. When the smoke barrier with sagging height of 1 m is spaced at 5 m intervals, the order of decreasing visibility of each monitoring point is A, B, J, and C, and the visibility of other monitoring points exhibits no change. At monitoring points A, B, J, and C, visibility begins to decline at 79 s, 92 s, 130 s, and 208 s, respectively. The visibility at monitoring points A and B finally stabilizes below 5 m, and the visibility at monitoring points J and C stabilizes at 5–10 m.

**Figure 7:**
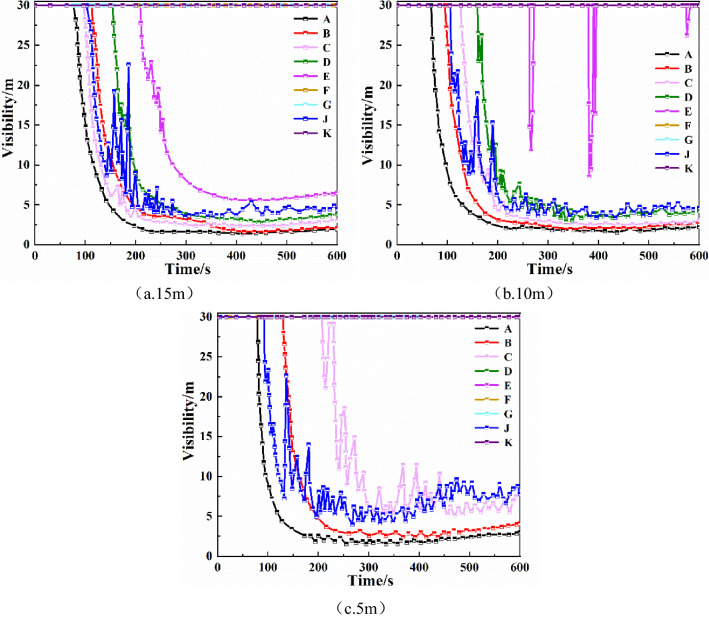
The visibility of each monitoring point of the smoke barrier with different spacing.
  From these results, it is shown that reducing the spacing between smoke barriers increases the visibility of monitoring points far from the fire source and delays the visibility drop at monitoring points farther away from the fire source, thereby facilitating the evacuation of people.
  In summary, the smaller the distance between smoke barrier, the higher the smoke layer height, the lower the temperature of the monitoring point, and the higher the visibility of the monitoring point farther from the fire source. Closer to the source of fire, the lower the height of the smoke layer, the lower the temperature of the monitoring point, the lower the visibility. Therefore, the best spacing for the smoke barrier is 5 m, which can have a more effective effect on the spread of smoke away from the fire source.

### Analysis of the impact of using a mechanical smoke extraction system combined with smoke barrier

Taking the middle fire source as an example, according to the above conclusion, we consider a smoke barrier with 5 m spacing and 1 m sagging height. Under the condition that the total smoke exhaust volume remains unchanged, the influences of the size, number, and arrangement of the smoke vents of a mechanical smoke exhaust system on the smoke exhaust effect are studied. As shown in Table 1, six different working conditions are set up. From the simulation results, the optimal setup scheme for a smoke exhaust system is determined. This provides a certain reference basis for configuring the smoke exhaust systems of underground malls.

**Table 1:** Mechanical smoke extraction system setup plan.

Cases	Number of smoke vents/(pc)	Smoke exhaust volume/(m^3^ s^−1^)	Smoke exhaust vent size/(m × m)
1	10 (single-row arrangement)	5	1 × 0.5
2	10 (single-row arrangement)	5	0.8 × 0.5
3	10 (single-row arrangement)	5	0.6 × 0.5
4	12 (single-row arrangement)	4.2	0.8 × 0.5
5	8 (single-row arrangement)	6.25	0.8 × 0.5
6	10 (double-row arrangement)	5	0.8 × 0.5

#### Analysis of the impact of smoke vent size

A mechanical smoke exhaust system is set up in the middle of the roof of the underground shopping mall, with 10 (single row setup) smoke vents evenly distributed and the smoke exhaust volume of each vent set to 5 m^3^/s. The dimensions of three smoke vent sizes are 1.0 m × 0.5 m, 0.8 m × 0.5 m and 0.6 m × 0.5 m^28^, the functioning of the mechanical smoke exhaust system during a fire is simulated, and the results are used to analyze the effect of different smoke vent sizes on the smoke exhaust effect under the condition that the total smoke exhaust volume remains unchanged.

1. Height of smoke layer at each monitoring point
  As can be seen from Fig. 8, with the mechanical smoke exhaust system, the smoke does not spread to monitoring points A, G, J, and K. The height of the smoke layer at monitoring points A, G, J, and K is kept at a high level. At monitoring points B, C, and E, the decline of the smoke layer is significantly delayed, and the height of the smoke layer rises slightly, but the degree of rise is not obvious. Comparing the changes in the height of the smoke layer at each monitoring point for three different smoke vent sizes, it is found that changing the smoke exhaust vent size does not have a significant effect on the height of the smoke layer at each monitoring point when the number of smoke vents and smoke exhaust volume remain the same.

**Figure 8:**
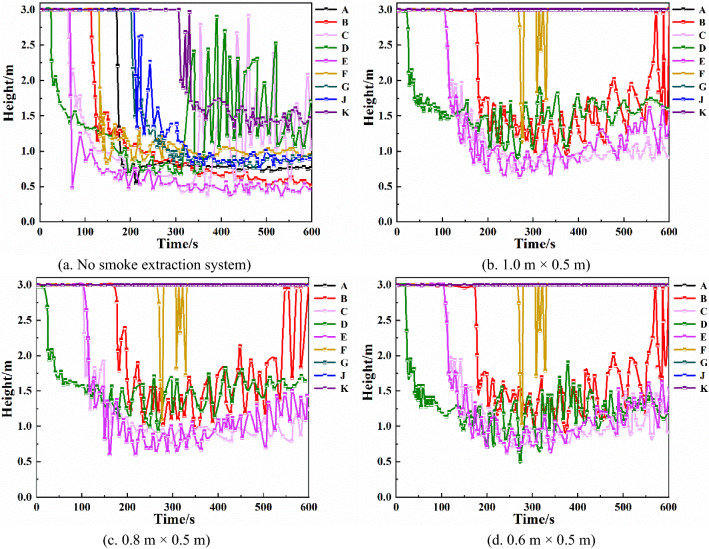
Smoke layer height at each monitoring point for different smoke exhaust vent sizes.
2. Temperature of each monitoring point
  As can be seen from Fig. 9, after adding the mechanical smoke exhaust system, the maximum temperature of monitoring point D increases, and the temperature of all other monitoring points decreases to different degrees. At monitoring points C and E, the rise in temperature is delayed by about 60 s, and the maximum temperature has decreased, and in about 420 s to below 100 °C. In 600 s, in addition to the nearest D monitoring point from the source of the fire, the temperature of each monitoring point are stable at 60 °C or less, the safety factor was greatly increased. Comparing the changes in temperature at each monitoring point of the three vent sizes, it is found that changing smoke exhaust vent size does not affect the temperature at each monitoring point when the number of smoke vents and smoke exhaust volume remain the same.

**Figure 9:**
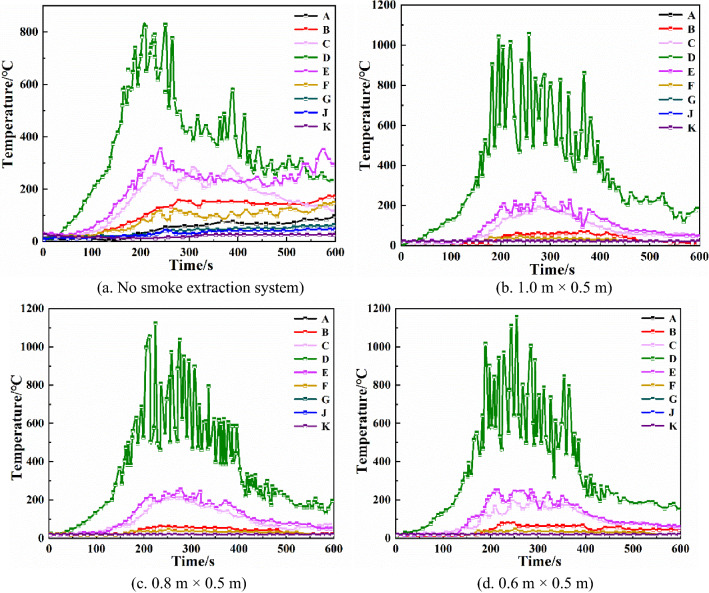
Temperature at each monitoring point for different smoke exhaust vent sizes.
3. Visibility of each monitoring point
  As can be seen from Fig. 10, with the mechanical smoke exhaust system, D monitoring point visibility changed little, B, C, E monitoring point visibility change time had been delayed, and in about 420 s there are different degrees of rise. Visibility rise at monitoring point B was the highest at more than 10 m. A, F, G, J, K monitoring points with very low smoke content, visibility was maintained at about 30 m. Comparing the changes in visibility at each monitoring point of the three vent sizes, it is found that changing the smoke vent size does not affect the visibility at each monitoring point when the number of smoke vents and smoke exhaust volume remain the same.

**Figure 10:**
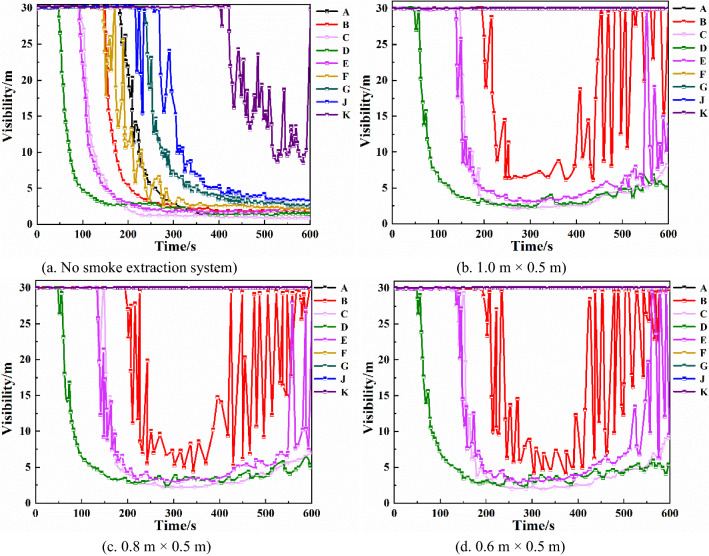
Visibility of each monitoring point with different smoke exhaust vent sizes.
  Through the above analysis, it can be seen that after adding the mechanical smoke exhaust system, the smoke exhaust effect is significantly improved. Changing the smoke vent size does not significantly affect the smoke exhaust effect.

#### Analysis of the impact of number and arrangement of smoke vents

Keeping the total smoke exhaust volume constant, the number and arrangement of smoke vents are changed, and the influence of both factors on the smoke exhaust effect is analyzed. A heat detector with a response temperature of 70 °C is set up in the upper part of the underground shopping mall. When the heat detector responds, the mechanical smoke exhaust system starts to work. The mechanical smoke exhaust system is set up as follows: each smoke vent size is 0.8 m × 0.5 m, 12, 10, and 8 smoke vents are evenly set up in a single row in the middle of the underground shopping mall promenade, and the smoke exhaust volume of a single vent is set to 4.2 m^3^/s, 5 m^3^/s, and 6.25 m^3^/s. Additionally, 10 smoke vents are placed in a double row, and the smoke exhaust volume of each vent is 5 m^3^/s^29,30^.

1. Height of smoke layer at each monitoring point
  From Fig. 11a–c, it can be seen that, with the same total smoke exhaust volume, increasing the number of smoke vents increases the height of the smoke layer at monitoring points B, C, D, E, and F to different degrees, and the most obvious height increase is at monitoring point C. Between 150 and 450 s, the smoke layer height at monitoring point C is around 0.5 m when the number of smoke vents is 8, around 1 m when the number of smoke vents is 10, and around 1.25 m when the number of smoke vents is 12. The smoke layer height at monitoring point F is most obviously increased, and when the number of smoke vents is 12, the height of the smoke layer is above 1.5 m. Increasing the number of smoke vents can effectively improve the height of the smoke layer.

**Figure 11:**
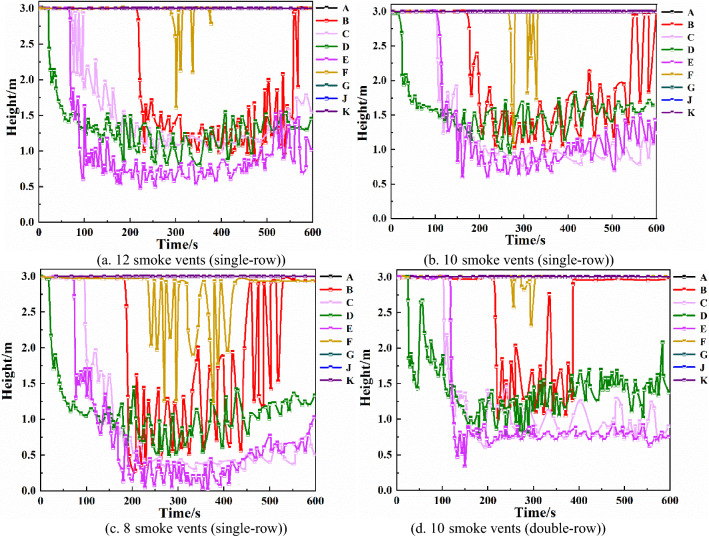
Smoke layer height at each monitoring point with different number and arrangement of smoke vents.
  Comparing Fig. 11b,d, it can be seen that when the smoke vents are placed in a double row, the height of the smoke layer at monitoring points B, C, and F is significantly higher than when the smoke vents are placed in a single row. The height of the smoke layer at monitoring point F is most obviously increased, by about 1.25 m, with the double row arrangement. Therefore, the double-row smoke vent arrangement is more effective than the single-row arrangement.
2. Temperature of each monitoring point
  From Fig. 12a–c, it can be seen that when the total smoke exhaust volume remains the same, changing the number of smoke vents does not have much effect on the temperature at monitoring points C, D, and E, which are closer to the fire source. Monitoring points B and F are affected the most. At monitoring points B and F, when the number of smoke vents is 8, the highest temperatures are 86 °C and 67 °C, respectively. When the number of smoke vents is increased to 10, the highest temperatures decrease by 24 °C and 22 °C, respectively. When the number of smoke vents is increased to 12, the highest temperatures decrease by 36 °C and 40 °C, respectively. Hence, increasing the number of smoke vents can effectively reduce the temperature of smoke at some of the monitoring points.

**Figure 12:**
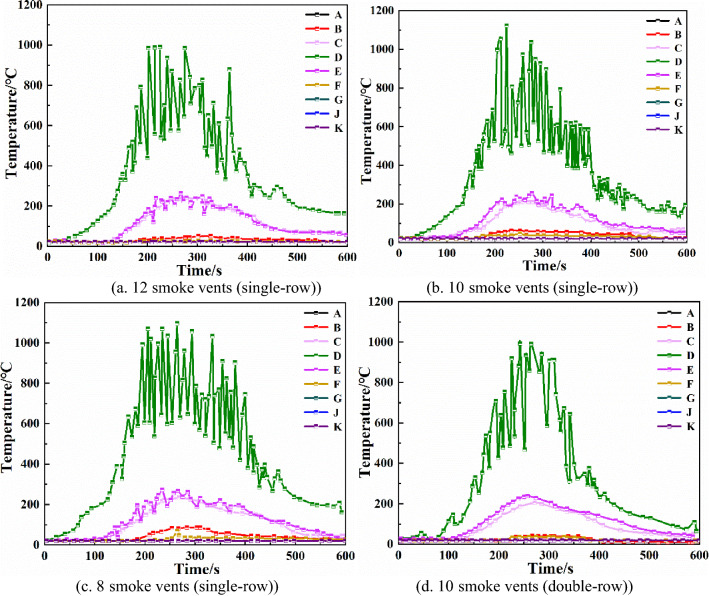
Temperature of each monitoring point with different number and arrangement of smoke vents.
  From Fig. 12b,d, it can be seen that when 10 smoke vents are set in a double row, the maximum temperature of monitoring point D decreases by 200 °C compared with the single-row arrangement, and the maximum temperature of monitoring point B decreases by about 20 °C. The double-row smoke vent arrangement is better than the single-row arrangement at reducing the temperature at the monitoring points.
3. Visibility of each monitoring point
  From Fig. 13a–c, it can be seen that when total smoke exhaust volume remains the same, the number of smoke vents does not have much effect on the visibility of monitoring points C, D, and E, which are closer to the fire source. The visibility of monitoring point B, which is a little farther away from the fire source, improves significantly. When the number of smoke vents is increased to 12, the visibility of B monitoring point is higher than 10 m, so the danger level is greatly reduced. Hence, increasing the number of smoke vents can effectively improve visibility.

**Figure 13:**
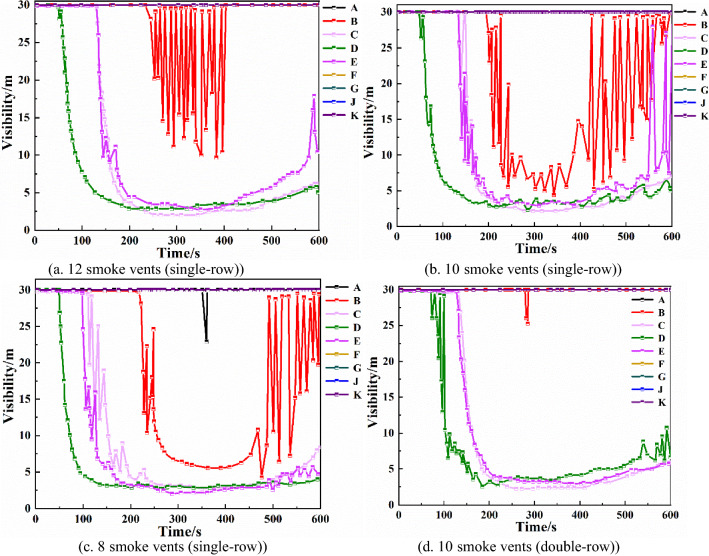
Visibility of each monitoring point with different number and arrangement of smoke vents.
  Comparing Fig. 13b,d, it can be seen that with the double-row smoke vent arrangement, the visibility decline at monitoring point D is delayed by 40 s, and the visibility at monitoring point B improves by about 20 m, indicating that the double-row arrangement results in significantly higher visibility than the single-row arrangement with lower smoke content.
  In summary, utilizing both a mechanical smoke exhaust system and smoke barriers can effectively reduce the temperature at each monitoring point and increase the height of the smoke layer and the visibility, which is conducive to the safe evacuation of occupants. In the case that the number of smoke vents and the total smoke exhaust volume remain unchanged, changing the size of smoke vents does not change the smoke exhaust effect significantly. In the case of constant total smoke exhaust volume, the best smoke exhaust effect is achieved when the number of smoke vents is 12. In the case of constant number of smoke vents, arranging smoke vents in a double row is more efficient than arranging smoke vents in a single row, and it greatly reduces the temperature at locations farther away from the fire source and improves the height of the smoke layer and the visibility.

## Conclusions

In this study, we investigate the influence of the sagging height and spacing of smoke barriers and the size, number, and arrangement of smoke vents in a mechanical smoke exhaust system on the effectiveness of smoke containment and exhaust via numerical simulation. The following main conclusions are drawn.

1. Within the range of 0.5–1 m of the sagging height of the smoke barrier, the higher the sagging height of the smoke barrier, the higher the maximum temperature of the monitoring point nearer to the fire source, the greater the height drop of the smoke layer, and the lower the visibility. The farther away from the fire source, the lower the temperature of the monitoring point, the higher height of smoke layer and the higher the visibility. When the sagging height of the smoke barrier exceeds 1 m, which is 1.2 m, the smoke-blocking effect is equivalent to 1 m.
2. The smaller the distance between smoke barrier, the higher the smoke layer height, the lower the temperature of the monitoring point, and the higher the visibility of the monitoring point farther from the fire source. Closer to the source of fire, the lower the height of the smoke layer, the lower the temperature of the monitoring point, the lower the visibility. The best spacing for the smoke barrier is 5 m, which can have a more effective effect on the spread of smoke away from the fire source.
3. Using both smoke barriers and a mechanical smoke exhaust system can provide more effective smoke control by combining smoke containment and exhaust. The size of the opening area of the smoke vent has no significant effect on the smoke exhaust effect. The best smoke exhaust effect is achieved when the number of smoke vents is 12, and the double-row arrangement of smoke vents is more efficient than the single-row arrangement.
4. Comprehensively considering the effectiveness and economy of smoke containment and exhaust, the optimal parameters for the combined smoke barrier and mechanical smoke exhaust system are as follows: smoke barriers with sagging height of 1 m and spacing of 5 m, and mechanical smoke exhaust system with 12 smoke vents in a double-row arrangement.

## Data Availability

The data and materials that support the results or analyses presented in our study are freely available.
